# Recent advances in understanding and managing Kallmann syndrome

**DOI:** 10.12703/r/10-37

**Published:** 2021-04-13

**Authors:** Du Soon Swee, Richard Quinton, Roberto Maggi

**Affiliations:** 1 Department of Endocrinology, Singapore General Hospital, Singapore; 2 Department of Endocrinology, Diabetes & Metabolism, Royal Victoria Infirmary, Newcastle-Upon-Tyne Hospitals, Newcastle-upon-Tyne, UK; 3 Translational & Clinical Research Institute, University of Newcastle-upon-Tyne, Newcastle-Upon-Tyne, UK; 4 Department of Pharmaceutical Sciences, Università degli Studi di Milano, Milan, MI, Italy

**Keywords:** Kallmann syndrome, congenital hypogonadotropic hypogonadism, minipuberty, GnRH

## Abstract

Many of the recent advances in our understanding of human reproductive biology and its genetic basis have arisen directly via the genetic investigation of patients with Kallmann syndrome and their families. The disease is characterised by the association of an isolated defect in the secretion (or, less commonly, action) of gonadotropin-releasing hormone (GnRH) and consequent infertility, with anosmia and potentially other associated non-reproductive features. GnRH-producing neurons are located in the hypothalamic brain region after a peculiar migration during embryonic life. To date, different genes affecting GnRH neuron development/migration have so far been implicated in Kallmann syndrome, but our knowledge of the genetic basis of the syndrome remains incomplete. From a clinical point of view, the disease has suffered from a lack of definitive diagnosis and treatment, and although progress has been made in terms of timely diagnosis and evidence-based treatment of patients, implementation remains inconsistent. These aspects will be discussed in this review, which examines new strategies for arriving at more evidence-based and patient-centred medical practice in Kallmann syndrome.

## Introduction

Kallmann syndrome (KS) and its normosmic variant—collectively known as congenital hypogonadotropic hypogonadism (CHH)—are principally characterised by isolated deficiency in the secretion or action of gonadotropin-releasing hormone (GnRH). Individuals with these conditions tend to present with failure to progress through puberty as well as infertility. Cryptorchidism and/or micropenis are also observed in a significant proportion of KS male infants—24–70.3% and 7.8–31.5% reported in cases series, respectively^1–4^—as a result of defective minipuberty. Besides anosmia, there are several other associated non-reproductive features, including midline facial, dental, and digit anomalies, hearing impairment, bimanual synkinesis, and renal anomalies^1,3^. Indeed, genetic evaluation may be required to help distinguish patients with severe forms of KS from those with milder forms of CHARGE^5,6^ and Waardenburg syndromes^7^. Intriguingly, reversal of hypogonadotropic hypogonadism may occur in up to one-fifth of cases^8^, albeit this may not be maintained long term^9^. Underpinning this disorder of such heterogeneous phenotypic manifestations is an immensely complex genetic architecture. Indeed, the varying penetrance and the interplay of pathogenic variants can give rise to a multitude of permutations in disease expression, even within the same kindreds. With less than half of cases having had an established genetic basis to date, there remains wide scope for work to be undertaken, and the impetus for these efforts is now provided by new diagnostic approaches, particularly next-generation sequencing techniques. This review aims to highlight recent developments in the understanding of GnRH developmental biology and its genetic pathogenesis and to provide clinical insights into the modern management of KS patients.

## The extraordinary journey of GnRH neurons

The reproductive defects of KS are due to the altered development of a neuronal network of just a few scattered hypothalamic neurons. This initiates and maintains reproductive function in mammals by coordinating the synthesis and pulsatile secretion of a neuroendocrine decapeptide, the GnRH. These neurons (about 1,500 in rodents) are not organised in a discrete brain nucleus but are instead distributed throughout the mediobasal hypothalamic region, making their investigation quite difficult, particularly in humans.

The origin and distribution of GnRH neurons are peculiar and conserved processes. In early embryos, GnRH neurons first appear in the nasal region (the olfactory placode) and, during foetal development, they undergo an extraordinary tangential migratory journey toward the brain hypothalamic region. These events have been studied extensively in several species^10,11^.

Until recently, information on GnRH neurons in humans has been scarce and obtained mainly at discrete embryonic/foetal stages^12,13^. However, more recently, by a combination of anatomical techniques, the first chronological and quantitative analysis of GnRH neuron origins, differentiation, and migration in the human foetal brain has become available^14^.

The overall number of GnRH immunoreactive neurons in humans is significantly higher than previously thought; actually, about 10,000 GnRH neurons are present in the whole brain during foetal life, with about 2,000 neurons located in the hypothalamus and 8,000 neurons distributed in widespread brain areas^14^, and that may account for the non-reproductive functions of GnRH^15^. Human GnRH neurons born in the olfactory region migrate (at gestational weeks 9–12) into the brain alongside fascicles of the terminal^16^ and vomeronasal nerves, sometimes collectively referred to as the accessory olfactory nerves. The role of alterations of olfactory/vomeronasal patterning on defective GnRH neuron migration and olfactory dysfunction is still not well clarified^17–19^. However, relatively normal reproductive functions, or a late-onset hypogonadism, have been described in sporadic patients with congenital olfactory agenesis with anosmia^20,21^; moreover, data from eugonadal patients with arrhinia show that human GnRH neurons may not entirely depend upon olfactory structures for their normal migration and function^22^. Studies on animal models unravelling the common origin of olfactory cells and a subpopulation of GnRH neurons from the neural crest^23^, an embryonic region that gives rise to the peripheral nervous system, bone, and melanocytes, may provide an additional interpretation of the syndromic association of hypogonadism and anosmia.

The recent setup of human pluripotent stem cell-derived human GnRH neurons might allow the identification of signalling pathways and gene regulatory networks involved in the human GnRH phenotype^24^.

## How many genes are involved in KS?

To date, more than 25 different genes affecting GnRH neuron development/migration have been implicated in KS^25^, but several aspects of their functions remain unclear^11^. To this purpose, different approaches of research are mainly used in the search of gene mutations in KS patients, or in patients with other diseases with clinical signs of KS, and *in vitro* validation, the characterization of genes/factors controlling GnRH neuron migration and detection of their mutations in KS patients, as well as the investigation of transgenic/knockout rodent models showing lack of fertility.

Some genes linked to KS are required for differentiation of the GnRH-secreting neurons, such as those coding for receptor–ligand pair fibroblast growth factor receptor 1/fibroblast growth factor 8 (FGFR1/FGF8), NMDA receptor synaptonuclear signalling and neuronal migration factor (NMSF, formerly nasal embryonic LH-releasing hormone factor, NELF), and heparan sulphate 6-O-sulphotransferase 1 (HS6ST1); other genes encode the essential signals for the correct migration of GnRH neurons, such as anosmin-1 (ANOS1), the ligand–receptor complex prokineticin 2/prokineticin receptor 2 (PROK2/PROKR2), and semaphorin 3A (SEMA3A)^26,27^.

**Figure 1.**
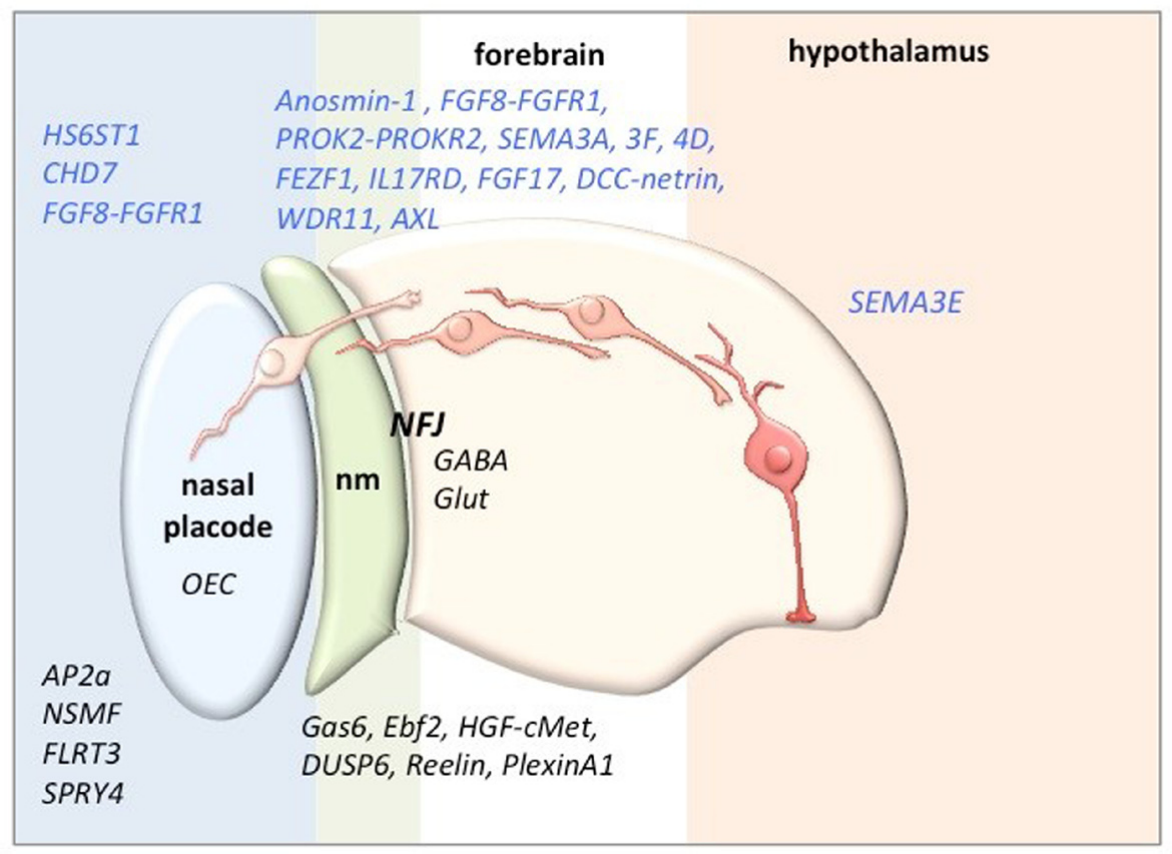
Some of the factors that have been confirmed (blue) or found to be candidates for pathogenetic factors of Kallmann syndrome and the location of their action along the migratory journey of gonadotropin-releasing hormone neurons (in shades of red) (adapted from 15). AXL, AXL receptor tyrosine kinase; CHD7, chromodomain helicase DNA binding protein 7; cMet, hepatocyte growth factor receptor; DCC, Deleted in Colorectal Carcinoma; DUSP6, Dual Specificity Phosphatase 6; Ebf2, early B cell transcription factor; FEZF1, FEZ Family Zinc Finger 1; FGF, fibroblast growth factor; FGFR, fibroblast growth factor receptor; FLRT3, Fibronectin Leucine Rich Transmembrane Protein 3; GABA, gamma aminobutyric acid; Gas6, growth arrest-specific 6; Glut, Glutamate; HGF, hepatocyte growth factor; HS6ST1, heparan sulphate 6-O-sulfotransferase 1; IL17RD, Interleukin 17 Receptor D; NFJ, nasal forebrain junction; nm, nasal mesenchyme; NSMF, NMDA receptor synaptonuclear signalling and neuronal migration factor; OEC, olfactory ensheathing cell; PROK, prokineticin; PROKR, prokineticin receptor; SEMA, semaphorin; SPRY4, Sprouty RTK Signaling Antagonist 4; WDR11, WD Repeat Domain 11.

Figure 1 summarises some of the factors affecting the development, differentiation, migration, and survival of GnRH neurons along their migratory journey to the hypothalamus that have been confirmed or are candidates for pathogenetic factors of KS. However, mutations of some of the candidate genes have been proposed to increase susceptibility to the disease in digenic or oligogenic forms.

Anosmin-1, the product of *ANOS1*, the first identified in KS^28^, was found to affect GnRH neuron migration and axon outgrowth; however, it may modulate olfactory ensheathing cell (OEC) maturation, an observation that may justify KS olfactory defects^29^. Anosmin-1 is also expressed in the urinary system from the early steps of kidney development (nephrogenesis), suggesting that the renal aplasia found in X-linked KS^20^ may result from the agenesis of the metanephros (the developing excretory organ of the foetus). Preliminary results seem to indicate that anosmin-1 might also affect the chemomigration of embryonic kidney cells (Maggi, in preparation), potentially underpinning its involvement in renal agenesis present in some men with X-linked KS^30^. Therefore, the embryonic expression and functions of anosmin-1 seem to faithfully recapitulate many defects found in KS.

Another characteristic of the emerging data on the genes involved in GnRH development/migration (as well as in KS) has been the identification of defined “pathological pathways”. For instance, anosmin-1 may exert its action through the modulation of FGF and semaphorin/VEGF receptor activity, and these interactions may clarify the similar KS phenotypes induced by individual mutations of such receptors^31^.

Again, the signalling pathway of the morphogenetic factor sonic hedgehog (Shh) could be another candidate in the defects of the olfactory and GnRH systems in KS^32^; accordingly, a loss-of-function variant of GLI3, encoding a transcription factor involved in Shh signaling^33^, and mutations of WD Repeat Domain 11 (WDR11), which modulates the proteolytic processing of GLI3^34^, have also been identified in KS. Additionally, several semaphorins—from a large family of guidance molecules implicated in neuronal development and GnRH neuron migration and survival—have been found to be mutated in KS^35,36^. Only an accurate interactome network analysis of the proteins already known to be involved in the KS phenotype^11^ will allow characterisation of possible overlays of different gene mutations or variants lacking a clearly demonstrable altered phenotype when present in monoallelic form^37^.

New evidence also underlines that genetic investigation cannot be limited to transcribed DNA sequences; intronic or intergenic mutations have recently been associated with the KS phenotype, suggesting that sequence variants located close to exon/intron boundaries should be experimentally investigated for pathogenicity^38–40^. Finally, a deletion in a long noncoding RNA, rhabdomyosarcoma 2-associated transcript (RMST), present on chromosome 12, has been identified in a KS patient, another finding that may bring innovative perspectives to the genetic diagnosis of KS^41^.

Although a growing number of genes related to KS are characterised, identifiable disease-causing genetic mutations are currently found in only about 40% of patients. Recent advances in next-generation sequencing technologies, with massively parallel sequencing of multiple samples^42^, and in bioinformatic analysis of the expression, in olfactory or GnRH cells, of genes causative of KS will allow the identification of new factors involved in GnRH neuron biology and in the disease^11,40^ to be screened for mutations in the remaining 60% of familial and sporadic KS cases, which are still waiting for a diagnostic framework.

## The clinical view

### Gaps in the care of individuals with KS

The immense advances in our understanding of the molecular mechanisms underpinning CHH could not have been achieved without the crucial contribution of patients and their families who gifted their DNA and phenotypic data for scientific research. Therefore, it is a matter of immense regret that timely clinical diagnosis and the overall quality of care of KS patients have barely improved over the past three decades, with the median age at which meaningful treatment is initiated across Europe unchanged at 18 to 19 years for both sexes^43^. Indeed, despite any significant genetic underpinning, the over threefold excess of male *versus* female patients recorded in most specialist centres suggests that many affected women may never achieve a definitive diagnosis, perhaps instead receiving empirical treatment in primary care or office-based gynaecology services.

CHH patients bear the consequences of clinicians missing key opportunities for diagnosis, treatment, and information sharing with them throughout their lives, which can be summarised as follows:

•   Failure to recognise neonatal “red flag” clinical features for CHH, leading to missed opportunities for making a definitive biochemical diagnosis within the postnatal minipuberty window, which in turn can lead to subsidiary missed opportunities to 44 a) deploy gonadotropin treatment in boys to achieve peno-scrotal enlargement and testicular maturation and descent, thereby obviating the need for surgical orchidopexy with its attendant risks of testicular damage or loss, and potentially also having a favourable impact on fertility potential in adult life, and b) initiate age-appropriate pubertal induction treatment with sex steroids in both sexes, rather than waiting for overt pubertal delay—with its attendant mental health issues^45^—to manifest.

•   Failure to properly contextualise pubertal delay associated with “red flag” clinical features as indicating CHH, rather than the more common scenario of constitutional delay^46^. Of note, kisspeptin stimulation recently proved to be a promising diagnostic tool in differentiating CHH and CDGP in a small cohort of adolescents, overcoming the limited discriminatory power of other tests including GnRH-stimulated LH, inhibin B, and genetic tests^47^, albeit larger-scale study would be necessary to validate the results.

•   Failure to initiate sex steroid treatment by 14 years of age in adolescents with pubertal delay, instead inappropriately deploying a “reassure and observe” strategy^48^.

•   Failure to ensure that adequate uterine maturation is achieved during pubertal induction of CHH (and other hypogonadal) girls, as well as adequate breast development. A proportionately smaller uterus in adult life is likely to reduce the chances of live birth after initially successful fertility treatment^49^.

•   Failure to recognise that one-third of CHH patients harbour a slightly milder form of GnRH deficiency/resistance^2^, such that the achievement of partial pubertal development (breast stage 3 or testes volume ≥4 mL) with a brief course of sex steroids may prompt inappropriate reassurance that “all will be fine” and, consequently, premature discharge of CHH adolescents from medical follow-up.

•   Failure to emphasise to both patients and their parents that sex steroid replacement will likely need to be for life in males or until the mid-sixth decade in females^50,51^.

•   Failure to explain to CHH patients and their parents that, whilst they are presently infertile, fertility can potentially be achieved with gonadotropin (or pulsatile GnRH) treatment^52^.

•   Failure to treat CHH males seeking fertility with combined gonadotropin therapy (FSH+hCG), instead first attempting hCG monotherapy, which is usually ineffective^53^.

•   Failure to treat CHH females seeking fertility with natural cycle gonadotropin (or GnRH) ovulation induction (with luteal support), instead directing them straight to more invasive and expensive superovulation-based IVF or ICSI. In the absence of other infertility factors, a good pregnancy outcome with ovulation induction (coupled with luteal support with progesterone of hCG and optimally timed sexual intercourse) is expected and should be offered as first-line therapy^54^. While pulsatile GnRH pump therapy is considered to be more physiological, it is inexplicably not readily accessible in many countries and has reduced efficacy in cases of gonadotroph resistance. Fortunately, gonadotropin therapy overcomes some of these obstacles, and careful dosing minimises the risk of multiple pregnancy and ovarian hyperstimulation^51^.

•   Failure to offer treatment to CHH females seeking fertility on the basis that “their AMH level is too low”, whereas low AMH is entirely to be expected in CHH females whose ovarian granulosa cells have never been exposed to FSH. Indeed, the low basal AMH level is not prognosticative of ovarian follicular reserve; it has been shown to reverse upon FSH stimulation in CHH females^55^, such that CHH females may be inappropriately counselled against proceeding with fertility treatments based on an ill-informed interpretation of the prognostic value of a low AMH level.

•   Failure to optimise oestrogen replacement in CHH females with oestradiol doses titrated according to serum oestradiol levels, bone density, and self-reported vaginal lubrication, instead treating these women empirically with fixed-dose ethinylestradiol-based combined oral contraceptive^56^.

## Deficient minipuberty underlies reproductive tract maldevelopment in KS

Whilst the majority of KS males manifest in adolescence or adulthood with pubertal failure or infertility, it is important for clinicians to bear in mind that the onset of dysregulated reproductive tract development began at the earliest stages of life. Indeed, deficiency in minipuberty of late gestation and early infancy is increasingly recognised as a key event in the disease course of KS, critically impacting on male external genital maturation and long-term fertility potential^48,57^. Recent literature has demonstrated the feasibility of intervention in early childhood, and similar principles could be applied to older individuals in an attempt to optimise spermatogenesis induction. Accordingly, formulating hormonal treatment that seeks to correct these fundamental developmental defects might hold the key to better reproductive outcomes.

During minipuberty, the surge in GnRH secretion creates the hormonal milieu necessary for the regulation of testicular descent into the scrotum. In particular, the Leydig cell-derived factors, including testosterone and insulin-like-3 peptide, modulate the inguinoscrotal phase of testicular descent and the eventual anchoring in the scrotal position. Concurrently, with the proliferation of Sertoli cells and seminiferous tubules, a sharp rise in serum inhibin B and anti-Mullerian hormone levels is observed, which typically peaks between 2 and 3 months after birth. These hormonal changes were recently well characterised in a large cohort of Danish male infants, demonstrating levels of circulating reproductive hormone levels that closely match those of adults^58^. Furthermore, in support of the key role minipuberty plays in ontogenesis, recent pooled data from Finnish and Danish birth cohorts have now strongly linked the postnatal changes in the position of testes (measured as testicular distance to pubic bone) and consolidation of intrascrotal position to both Leydig and Sertoli cell function^59^.

Consequently, the deficiency of GnRH secretion and/or action in KS is highly disruptive to the developmental process, leading to phenotypic manifestation of cryptorchidism and micropenis in infants. In addition, clinically less apparent but equally important is the diminished population of Sertoli and germ cells resulting from reduced FSH signalling. These early childhood events have far-reaching consequences on fertility potential; such severely affected individuals (i.e. complete CHH), who represent around two-thirds of CHH males in a recent series^3^, would classically present with pubertal failure with testicular volumes of <4 mL. These men consistently experience a worse response to spermatogenesis induction by gonadotropin therapy compared to men with partial CHH. Interestingly, research conducted in rhesus monkeys showed that the switching-off of the pituitary–testicular axis with a GnRH agonist during the neonatal period, albeit temporarily, led to stunted testicular growth and reduced sperm production later in life during puberty^60^. This underscores the integral role of minipuberty in the development of long-term testicular function.

## Replicating minipuberty through gonadotropin treatment: the key to optimising outcomes 

The feasibility of hormonal therapy in KS male infants in the replication of minipuberty to stimulate physiological penile and testicular development has been demonstrated. Papadimitriou and colleagues recently reported their experience with combined gonadotropin treatment in a cohort of 10 male neonates and infants affected by bilateral cryptorchidism and micropenis due to absent minipuberty, as confirmed by repeatedly low serum levels of gonadotropins and testosterone sampled in the first 3 months after birth^61^. These children received recombinant LH (75 IU of lutropin alfa) and FSH (150 IU of follitropin alfa) delivered daily via subcutaneous injection. Following 3 months of treatment, significant improvement in inhibin B, AMH, and testosterone levels was observed, signifying the restoration of testicular function under gonadotropin stimulation. Moreover, over the course of treatment, testicular descent into the scrotal position was successfully induced in all cases, along with doubling of penile length from a median of 2.0 to 3.8 cm. Similar outcomes were observed in an earlier study involving the use of recombinant LH and FSH in five male infants with CHH, where micropenis improved by an average of 30 mm after 3–6 months of treatment, with concurrent testicular descent induced in two of the four cryptorchid boys^62^. By this early treatment strategy, testicular growth is restored in a timely fashion that closely matches the tempo of minipuberty, which is hopeful for better reproductive prospects in adulthood, although data remain lacking at this point. In the only long-term follow-up report of three CHH boys (at an average age of 11 years) who previously received rh-FSH and testosterone therapy in infancy, aside from unstimulated inhibin B level, there was no information on their clinical or spermatogenic response to pubertal induction^63^.

In contrast, FSH in combination with exogenous testosterone instead of LH did not appear to exert any demonstrable beneficial effect on testicular positioning in CHH infants, which echoes historic data from adults in respect of induction of spermatogenesis^64^. In a retrospective study of five infants treated with such a regimen, cryptorchidism failed to correct in three while testicular ascent occurred in the other two, thereby necessitating bilateral orchidopexy in all, despite having elicited a good response in both Sertoli cell function and penile growth^63^. This would indicate the importance of synergistic action of both FSH and LH in creating the hormonal milieu necessary for testicular descent and consolidation of intrascrotal position of testes. In part, paracrine effects of Leydig cell-derived androgens are dependent on their high intratesticular concentrations, which are not attainable by exogenous testosterone therapy. It should be emphasised that LH therapy in early childhood does not lead to spermatogenic activity because human Sertoli cells do not express androgen receptors until around 5 years of age.

Building on the key biological principles of testicular ontogenesis in infancy, evidence has emerged to support the use of such a hormonal treatment strategy in adolescents and adults to optimise reproductive function. In particular, those who exhibit a severe degree of GnRH deficiency would benefit most from minipuberty-like hormonal intervention through a period of FSH priming prior to the introduction of LH/hCG therapy that aims to facilitate maximal proliferation of immature Sertoli cells under FSH signalling, similar to the wave of growth normally present during minipuberty, before being subjected to LH-mediated terminal differentiation. Accordingly, in a randomised controlled trial of 13 non-cryptorchid CHH adult men with pre-pubertal testicular volume (<4 mL), the use of unopposed FSH (4 months) prior to 24 month GnRH replacement therapy showed promising data in spermatogenic outcomes compared to subjects who received GnRH therapy alone, including higher sperm counts and shorter time to sperm appearance^65^.

Indeed, the quest for novel treatment strategies seeks to address the treatment gaps of more commonly employed traditional regimens in severe CHH individuals. With hCG monotherapy, very prolonged treatment duration is typically required before a response, if any, may be observed: in a study of 110 CHH men, sperm production among those with a mean baseline testicular volume of <4 mL was non-evident until a median treatment duration of 3.5 years in the responders (the proportion of responders to non-responders was unclear from the report)^66^. Additionally, of those who did not experience any improvement in testosterone levels during treatment, comprising 5% of the entire cohort, they had either pre-pubertal testicular volume or cryptorchidism. With regard to combined gonadotropin treatment, Liu *et al*. recently reported a 67% spermatogenesis induction rate in a cohort of 183 non-cryptorchid CHH men with a mean baseline testicular volume of 2.2 mL, whereas an even lower spermatogenic rate of 50% was recorded in a separate group of 40 men with a history of cryptorchidism (baseline testicular volume of 1.6 mL)^67^. Intriguingly, orchidopexy was not associated with a more favourable outcome as would be usually expected for non-CHH aetiologies. These data further support the need for targeted intervention to mitigate the sequelae of defective minipuberty.

On the other hand, individuals with milder (partial) CHH tend to experience more favourable spermatogenesis induction outcomes that are comparable to those with acquired hypogonadotropic hypogonadism. In a retrospective study by Ortac and colleagues, conventional gonadotropin therapy (addition of FSH to hCG when azoospermia persists for ≥6 months) in their cohort of 112 men with a mean baseline testicular volume of ≥4 mL (5–5.3 mL) achieved a spermatogenic rate of 85%^68^. Therefore, such individuals could potentially be treated with a less-intense approach of hCG monotherapy before adjunctive FSH is considered, although combined treatment from the outset would also be valid. More importantly, this highlights the need to tailor gonadotropin therapy according to the clinical severity of GnRH deficiency in order to achieve the most desirable results while balancing the commitment (time, effort, financial, and emotional) required of patients to persevere with a potentially long treatment process.

As alluded to earlier, orchidopexy does not appear to ameliorate the response to fertility induction in CHH-associated cryptorchidism, which affects two in five CHH males based on composite data from several published series^44^. This is not surprising, as the underlying biological defect is primarily hormonal owing to impaired minipuberty, rather than purely anatomical in nature. Fundamentally, orchidopexy cannot address the lack of gonadotropin-mediated transformation of gonocytes into Adult dark (Ad) spermatogonia in cryptorchid testes in infancy, which is a key determinant of fertility in adulthood. Even in unilateral cryptorchidism, the risk of contralateral testis possessing similarly unfavourable spermatogonia development is significant^69^. Another issue with surgery is the inherent risk of tissue damage during manipulation that will further reduce the viability of the spermatogenic tissue. Both the smaller testes and shorter spermatic cords characteristic of neonatal CHH testes make them harder to mobilise and thus more prone to surgical misadventure. Hence, in the management of adolescents or adults with CHH who present late with untreated cryptorchidism, it could be worthwhile exploring the potential for gonadotropin therapy to stimulate testicular enlargement and descent in the first instance before resorting to surgical correction, similar to experiences in infant CHH. Such a possibility is borne out of our observation of a 19-year-old man whose testis descended following a course of preoperative gonadotropin therapy that was originally intended to facilitate (rather than obviate) orchidopexy^70^.

## Conclusions

Over the past three decades, major advances in our understanding of human development, reproductive biology, and neuroendocrine physiology, as well as of genetics and heritability itself, have arisen directly as a result of the genetic investigation of KS patients and their families. Given that our knowledge of the genetic basis of KS remains incomplete, it is likely that further major insights will continue to accrue with each new gene discovery.

Nevertheless, there has been little progress in improving patients’ experiences in terms of timely diagnostics and evidence-based treatments over the course of their journey from birth to (potential) reproductive maturity. Existing diagnostic and therapeutic strategies are not being reliably implemented because of medical practice in this area being more “habit based” than evidence based or patient centred.

Ultimately, almost every patient with any condition will necessarily present to a generalist physician in the first instance. However, with sexual and reproductive medicine only superficially appearing in medical school curricula, achieving a high-quality “first contact” with appropriate and timely onward referral to specialist care is not straightforward.
